# Traumatic Lumbar Interdural Cyst with Intradural Expansion and Compression of the Cauda Equina: Case Report and Surgical Video

**DOI:** 10.7759/cureus.4824

**Published:** 2019-06-04

**Authors:** Salah G Aoun, Aaron R Plitt, Tarek Y El Ahmadieh, Mazin Al Tamimi, Tony Whitworth

**Affiliations:** 1 Neurosurgery, University of Texas Southwestern Medical Center, Dallas, USA

**Keywords:** intradural cyst, interdural cyst, cauda equina compression, traumatic spine cyst, surgical video

## Abstract

Intradural arachnoid cysts are common entities that can be congenital, or caused by infectious, inflammatory, or even traumatic processes. However, true "inter"-dural cysts formed between the two lamellae of the lumbar dura without any fistulous arachnoid connection are rare. We present the case of a post-traumatic interdural cyst formation of the lumbar spine that compressed the roots of the cauda equina causing acute unrelenting pain. The cyst walls were formed by the true dural layers, and the cavity was filled with blood degradation products without any arachnoid connection to the subdural space. A commented video that details the diagnostic and surgical aspects of this case, alongside intraoperative footage is provided.

## Introduction

Intradural cyst of the spinal cord usually consist of spinal fluid-filled diverticula that can be found incidentally, or result in the compression of the nerve roots of the cauda equina, or of the spinal cord itself. While some can be congenital, other etiologies include inflammatory or infectious processes, or even traumatic events [1-3]. These events usually result in folds and adhesions in the arachnoid layer, or in a ball-valve phenomenon through an arachnoid fistula that can generate large bubbles, and compress the functional intradural components. However, a true cystic formation between the dural layers, also called interdural cyst, is very rare, with a handful of cases described in the literature [3-7]. To our knowledge there has only been one instance of traumatic interdural cyst that was reported in the literature, but that was due to a fistulous connection with the arachnoid layer of the nerve root sleeve. We report a case of post-traumatic lumbar interdural cyst that likely resulted from a hematoma formation between the two dural layers and required surgical drainage and marsupialization.

## Case presentation

**Figure 1:**
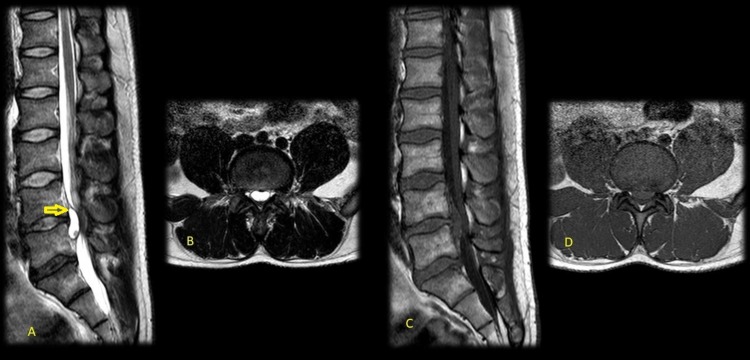
Preoperative Magnetic Resonance Imaging of the Lumbar spine A: Sagittal T2 sequence, B: Axial T2 Sequence, C: Sagittal T1 sequence D: Axial T1 sequence This magnetic resonance imaging sequence shows a lesion (yellow arrow) that is hyperintense on T2 sequences and isointense on T1, with contents that resemble spinal fluid.

A previously healthy 37-year-old man experienced an acute onset of back pain radiating to bilateral S1 territories after a soccer match while bending over to tie his shoes. His pain worsened over the course of 6 months which prompted a magnetic resonance scan (MRI). The MRI showed a cystic lesion ventral to the thecal sac at L4-5 which was compressing the roots of the cauda equina (Figure 1).

He was taken to the operating room, where a midline durotomy revealed a bulging cystic mass compressing the nerve roots (Video 1).

**Video 1:** Operative Video Operative video showcasing the clinical case, imaging characteristics, and intra-operative surgical resection footage of the lumbar spinal cyst

**Figure 2:**
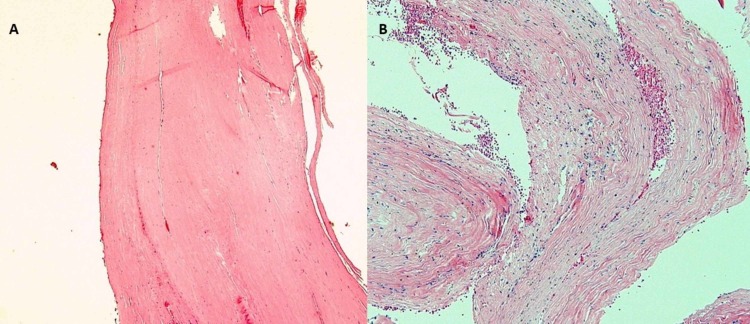
Pathology Slides of the Resected Lumbar Cyst A: Hematoxylin Eosin slide showing the cyst wall which was consistent with collagenous tissue forming dura B: Hematoxylin Eosin slide showing the content of the cyst which was consistent with fibrous connective tissue and blood

The mass appeared to be intradural. It did not originate from the disc space and had no ventral tract or fistulous connection. The wall of the cyst was sharply opened and had the consistency and feel of dura. The cyst was filled with motor-oil fluid that resembled the old blood. The cavity was lined with fibrous material that was sent for pathology with part of the wall (Figure 2).

The cyst was completely marsupialized and then cauterized. The patient did well after surgery and was discharged home on post-operative day two neurologically intact. His symptoms were completely resolved.

## Discussion

True interdural cysts of the spinal cord are rare entities [3]. Inflammation or infection can result in arachoiditis with the resulting sequestration of the spinal fluid in chambers that can grow and expand with time. On the other hand, traumatic injury of the spine can result in the avulsion of the neural tube at the level of the nerve root sleeves, and lead to intradural arachnoid cyst formation, or even interdural expansile spaces, but these are usually the product of a fistulous arachnoid connection. In our patient, there was no evidence of a connection diverting spinal fluid from the intradural space to the cyst. The cyst was also perfectly formed by leaflets of the dura, and was filled with motor-oil fluid and had traces of hemosiderin, which led us to believe that the trauma caused by trunk flexion must have ruptured a small dural vessel, leading to the formation of an acute hematoma between the ventral dural lamellae. We elected to fenestrate the cyst widely and cauterize its contents to prevent the creation of a one-way spinal fluid valve leading to the recurrence of the cyst, or the herniation and sequestration of the nerve roots of the cauda equina into the dural pouch which could lead to neurological deficits. 

## Conclusions

This is the first report of a traumatic lumbar interdural cyst with compression of the cauda equina roots. Treatment of these lesions requires careful dissection of the intradural space. Adequate cyst fenestration is required in order to prevent recurrences. 
